# Is Nursing a Profession? Some Obvious Omissions

**Published:** 1919-02-22

**Authors:** Gladys M. E. Leigh


### THE EDITOR'S LETTER-BOX.

### Is Nursing a Profession? Some Obvious Omissions.

*To the Editor of* The Hospital.

Sir,—Your correspondent "Ierne," in her dissertation as to whether "nursing" should, or should not, be considered a profession, overlooks some obvious facts. (1) Candidates desirous of entering a profession are required to produce proof of their educational status before they are admitted as students. Surely the time has arrived for the recognised Training Schools for Nurses of Great Britain and Ireland to insist that would-be probationers should produce the same guarantee. If this omission were rectified it would be a big step towards raising the social status of British nurses. (2) The training necessary to produce a proficient nurse is unique. It is necessary that a woman, who later may be called upon not only to nurse the sick, but to administer successfully a hospital ward or control a large female staff, should be cognizant of the quickest and most direct methods of obtaining the best results. For this purpose not only theoretical but practical knowledge is necessary. Of late years there has been a generous curtailment of the probationer's period of drudgery. (3) When "Ierne" suggests that the age limit should be reduced to eighteen, she ignores the fact that the long vacations granted to most students making it possible for them to travel and to develop tastes beyond their stereotyped curriculum are not possible for probationers.—Yours faithfully,

Gladys M. E. Leigh.

